# Optimizing Maize Yield With Hybrids Tolerant of High Plant Density in West and Central Africa

**DOI:** 10.1002/pei3.70046

**Published:** 2025-03-27

**Authors:** Wendm Ygzaw, Beatrice Elohor Ifie, Priscilla Francisco Ribeiro, Gloria Boakyewaa Adu, Eric Yirenkyi Danquah, Samuel Kwame Offei, Pangirayi Bernard Tongoona

**Affiliations:** ^1^ Department of Plant and Horticultural Sciences, College of Dryland Agriculture and Natural Resources Mekelle University Mekelle Ethiopia; ^2^ West Africa Centre for Crop Improvement (WACCI) University of Ghana Accra Ghana; ^3^ Institute of Biological, Environmental and Rural Sciences Aberystwyth University Aberystwyth UK; ^4^ CSIR – Crops Research Institute Kumasi Ghana; ^5^ CSIR‐Savanna Agricultural Reseatch Institute Tamale Ghana

**Keywords:** F_1_ hybrid, maize, multiple environments, plant density

## Abstract

The use of high plant density tolerant maize hybrids was one of the most successful interventions that boosted maize yield in the developed world. However, very little research has been conducted in the improvement of maize for high plant density tolerance in West and Central Africa (WCA). This study aimed to identify high plant density‐tolerant maize hybrids adapted to multiple environments. Forty‐eight maize hybrids were evaluated under three plant densities (low = 53,333, medium = 66,666, and high = 88, 888 plants ha^−1^). The experiment was conducted in four different environments in Ghana using 8 × 6 alpha lattice design with split plot arrangement. Plant density was the main plot and hybrids arranged in incomplete blocks within each plant density. The results revealed that the relative grain yield performance of the genotypes was dependent on plant density. Optimum plant density for the hybrids varied with growing environments. The highest grain yield of 9.5, 9.2, and 8.6 t ha^−1^ were obtained from the high plant density in Legon (minor season), Fumesua, and Legon (off‐season), respectively, and it was 26.7%, 22.7%, and 30% increase in comparison to the respective yield under the low density. F_1_ hybrids M131 × CML16, CML16 × TZEI1, CML16 × 87,036, TZEI387 × CML16, and ENT11 × 87,036 are good candidates for high‐density planting in high‐yielding environments. Grain yield performance of the maize hybrids was highest under high plant density for most of the growing environments. Thus, implementing high‐density planting for maize hybrids could be one of the options for increasing maize yield in West and Central Africa.

## 1 Introduction

Maize is an important cereal crop that ranks third after wheat and rice in its importance worldwide (Verheye 2010). It is the most widely cultivated and most important crop in Sub‐Saharan Africa. In Ghana, it is the most widely cultivated crop with the highest area coverage next to cocoa (Ministry of Food and Agriculture (MoFA) 2021). However, the average yield of maize for the year 2021 in Africa and Ghana was 2.22 and 2.50 t ha^−1^, respectively, which is much lower than the world average of 5.88 t ha^−1^ (FAOSTAT 2024). Several factors are attributed to the low yield obtained in Ghana including pests and diseases, abiotic stress, and agronomic practices.

Globally, maize yield increased by more than double from 1961 to 2002 (Duvick 2005). Increased plant density was a single major change that had boosted the yield of maize, although different improved agronomic practices, improved varieties, and hybrids had also contributed to the yield increase (Fischer et al. 2014). The optimum plant density for maize depends on the nature of the genotype, agronomic practices, and the growing environment (Sangoi 2000). Plant density above the optimum for a particular genotype under a specific environment causes yield reduction due to increased competition between individual plants for necessary resources (Matthijs and Wu 2000; Sangoi 2000).

In North America, maize is planted at an average density of 90,000 plants ha^−1^ or more as opposed to the 30,000 plants ha^−1^ which was practiced eight decades earlier (Assefa et al. 2018). It was also indicated that some varieties can give the highest yield when planted at 133,000 plants ha^−1^ in the USA (Mansfield and Mumm 2014). The good response of maize to increased plant density is partly attributed to its low tillering capacity (Fischer et al. 2014). This low tillering capacity makes it difficult for maize to fill the extra space when planted under low plant density (Sangoi 2000). However, old maize hybrids do not perform well under the contemporary high plant density (Fischer et al. 2014; Matthijs and Wu 2000; Sangoi 2000), indicating the role of genetic improvement in high plant density tolerance.

The better performance of new hybrids under high plant density indicates the possibility of exploiting the genetic basis of maize for improving high plant density tolerance. Moreover, the gradual and simultaneous increase of plant density and maize yield in the developed world (Duvick 2005; Fischer et al. 2014) indicates the possibility of increasing maize yield by improving the high plant density tolerance of maize in the developing world. Plant spacing of 50 cm between plants and 75 cm between rows, with two plants per hill, or plant spacing of 25 cm between plants and 75 cm between rows, with one plant per hill (i.e., 53,333 plants ha^−1^) is the common practice of maize production in West and Central Africa. Adu et al. (2014) recommended a higher plant density of 66,666 plants ha^−1^ for early maturing varieties in Ghana.

Globally, the demand for maize is expected to increase by more than twofold in the coming 30 years due to an increase in population and changes in feeding habits (Aramburu‐Merlos et al. 2024). The increased demand should be fulfilled either by expanding the arable lands or by increasing the yield per unit area. The second alternative should, undoubtedly, be the preferred way of increasing maize production considering that arable lands are limited. In view of the improvements made in the developed world, there is a huge potential for increasing the yield of maize in Sub‐Saharan Africa by introducing superior maize hybrids that are tolerant to high plant density.

Despite its potential for increasing yield, only a few attempts have been made in Sub‐Saharan Africa related to high plant density tolerance of maize, and most of them were either focused on the identification of optimum plant density for existing maize hybrids, or they were tested only on a few locations with a limited number of hybrids (Ajayo et al. 2021; Buah et al. 2009; Kamara et al. 2020; Sibonginkosi et al. 2020; Worku et al. 2020). In most of these studies, 66,666 plants ha^−1^ were considered as high plant density which is, actually, very low compared to the 90,000 plants ha^−1^ average plant density in North America. Moreover, studies which were conducted in Ethiopia and Nigeria have integrated different levels of nitrogen fertilizer with the plant density experiment (Ajayo et al. 2021; Worku et al. 2020), which might obscure the real tolerance of the hybrids to high plant density. In Ethiopia, the highest yield was obtained from 90,900 plants ha^−1^ with the application of 360 kg ha^−1^ of nitrogen (Worku et al. 2020). However, the experiment was conducted only in one location, two seasons, and one variety. Testing a substantial number of hybrids in many environments by applying similar agronomic management and similar fertilizer rates to all plant densities would provide better results when selecting maize hybrids that are tolerant to high plant density. Therefore, this study was carried out to develop maize hybrids from existing inbred lines and identify hybrids that perform better under high plant density across different locations in Ghana and to determine the effect of high plant density on important agronomic traits of maize.

## 2 Materials and Methods

### 2.1 Planting Materials

Forty‐five single cross F_1_ hybrids of maize developed from a half diallel cross of 10 inbred parents, one three‐way cross commercial check (PAN53), and two other single cross F_1_ hybrids were used in this study. The two additional single cross F_1_ hybrids were added to make the number suitable for alpha lattice.

### 2.2 Description of the Study Area

The experiment was conducted in four different environments in Ghana in 2018 which included two seasons (minor season and off‐season with irrigation) in Legon, the major season in Fumesua, and the rainy season in Nyankpala. The three different locations were selected because of the differences in their agro‐ecological zones (Table 1). The sites also differed in their soil properties (Table 2). The same, fully fenced, experimental site was used in Legon for the minor season and off‐season experiments. The difference between the minor season and the off‐season experiments was mainly the temperature and method of water supply. Thus, the two different season experiments in Legon were considered as two different growing environments. Drip irrigation was used to water the plants in Legon during the off‐season before the onset of rainfall and when there was a shortage of rainfall in the minor season. The agro‐climatic conditions of the study sites are summarized in Table 1.

**TABLE 1:** Agro‐climatic conditions of the study sites.

Environment	Geographic location	MAR (mm)	MAT (°C)	MT during experiment (°C)	Agro‐ecology	Altitude (m.a.s.l)
Legon‐minor season	5°39′37″ N 0°11′28″ W	800	26.65	28.54	Coastal savanna	81
Legon‐off season	5°39′37″ N 0°11′28″ W	800	26.65	27.75	Coastal savanna	81
Fumesua	6°42′53″ N 1°32′23″ W	1345	26.1	27.54	Transitional forest	293
Nyankpala	9°23′28″N1°00′28″ W	1075	28	27.93	Guinea savannah	180

Abbreviations: m.a.s.l, meters above sea level; MAR, mean annual rainfall; MAT, mean annual temperature; MT, mean temperature.

*Source:* Abbam et al. (2018); AccuWeather (2019); Ghana Meteorological Agency (GMet) (2019); Ghana Statistical Service (2014a); Ghana Statistical Service (2014b); Kwame Nkrumah University of Science and Technology (KNUST) (2019); Ministry of Food and Agriculture (MoFA) (2016); Nkrumah et al. (2014); Savanna Agricultural Research Institute (SARI) (2019), Tontoh (2011).

**TABLE 2:** Soil Chemical and Physical Properties of the Study Sites.

Site/Soil properties	Legon	Nyankpala	Fumesua
pH (H_2_O_1:1_)	4.23	3.5	4.9
EC (μs/cm)		135.3	161.6
Avail P (mg/kg)	58.58	8.63	129.75
N (%)	0.05	0.046	0.025
C (%)	1.28	0.49	1.04
K (Cmol/kg)	0.4	0.02	0.06
CEC (Cmol/kg)	3.83	0.223	1.02
Particle Size (%)			
Sand	64.07	81.25	72.77
Silt	7.6	6.25	3.47
Clay	28.33	12.5	23.75
Texture	Sandy Clay Loam	Sandy Loam	Sandy Loam

Composite soil samples taken from at least nine random spots in each site were analyzed for selected soil properties in the laboratory of the Department of Soil Science, University of Ghana. The results of the soil analysis are presented in Table 2.

### 2.3 Plant Densities and Field Evaluation

The study had three factors, namely environment, genotype, and plant density with four, 48, and three levels, respectively. Forty‐eight F_1_ maize hybrids were evaluated under three different plant densities (low = 53,333, medium = 66,666, and high = 88,888 plants ha^−1^) in the four environments. The experimental design was an alpha lattice in a split plot arrangement. Each of the three plant densities was arranged in separate parallel blocks (main plots of split plot design) where each of these main plots was divided into eight incomplete blocks, and each incomplete block contained six plots. Each plot was then assigned with one random hybrid (8 × 6 alpha lattice within each plant density). A two‐row plot of 4 m length each was used as an experimental unit. The inter‐row spacing was kept at 75 cm for all plant densities, and thus, the intra‐row spacing for the low, medium, and high plant densities were 25, 20, and 15 cm, respectively, resulting in each experimental plot being 6 m^2^. The plants at the start and end of each row were excluded during data collection to avoid border effect. Thus, the effective plot was 5.25, 5.4, and 5.55 m^2^ for the low, medium, and high plant density, respectively. Two seeds per hill were planted and later thinned to one plant per hill 14 days after planting. The treatments were replicated two times.

Standard cultural practices including weeding and irrigation (when needed) were undertaken for each plot. Similarly, fertilizers (i.e., compound N P K and urea) were applied for each plot equally. The rate of fertilizer applied was 60 kg ha^−1^ P, 60 kg ha^−1^ K, and 120 kg ha^−1^ N. All the P and K and two thirds of the N fertilizers were applied 2 weeks after planting while the rest of the N fertilizer was applied 6 weeks after planting. A mixture of insecticides of Attack which contained 5% Emamectin Benzoate, and Hercules containing Fipronil (50 g l^−1^) was applied two times a week to control termites and a fall army‐worm infestation, until the plants started tasseling. Roundup, a non‐selective herbicide, containing 360 g l^−1^ of glyphosate, was applied pre‐planting. After seedling emergence, hand weeding was carried out. Manual harvesting was carried out at maturity.

### 2.4 Data Collected

Phenotypic data were taken using modified procedures described in Sangoi et al. (2001), Sharifi and Gholipouri (2009), Alkhalifah (2013), Mansfield and Mumm (2014), and Al‐Naggar et al. (2016) (Table 3). Plants at the start and the end of each plot (border plants) were not included in the data. Only plants that were properly bordered (spaced) were included in the data, and the effective plot size was adjusted.

**TABLE 3:** Methods of data collection on selected traits.

Trait	Method of data collection
Number of upper leaves per plant	Counting the number of leaves above the upper ear
Chlorophyll content	Taken from the leaf above the upper ear using SPAD 502 Chlorophyll Meter at the dough stage of the plant
Leaf angle	The angle between the vertical stem and the leaf was determined using an 11 cm length of protractor
Plant height(cm)	The length from the ground to the lowest branch of the panicle
Ear heights	The length from the ground to the node where the upper ear emerged
Stem diameter	The diameter of the second internode (from the ground), determined using a digital Vernier caliper
Percent root lodging^a^	(total number of roots leaning > 45°)/(total plant stand) ×100
Percent stem lodging^a^	(total number of broken stems below the upper ear)/ (total plant stand) × 100. Determined 1 day before the harvesting date
Tassel size	Determined as (tassel branch number × tassel branch length); tassel branch length was determined as the average length of the lowest, middle, and upper tassel branches
Days to 50% anthesis and 50% silking^a^	Number of days from planting to pollen shedding and visible silk in 50% of the plants, respectively
Anthesis‐silking interval (ASI)^a^	Number of days between 50% anthesis and 50% silking.
Days to physiological maturity^a^	Number of days from planting until the husk of 90% of the plants changes to brown
Percentage of barren plants^a^	(total number of plants bearing no ear or no kernel in their ears)/(total plant stand of the effective plot) × 100
Ears per plant^a^	(the total number of ears)/(total number of plants)
Ear length and filled earl length	The length of the whole ear and the length of the portion of the ear filled with kernels, respectively
Ear diameter	The diameter of the center of the ear, determined using a Vernier caliper
Number of kernels per ear	Rows per ear × kernels per row
Hundred kernel weight	Weight of 100 randomly sampled seeds
Shelling percentage	(grain weight/unshelled ear weight) × 100
Grain yield (t ha ^−1^) at 15.5% moisture content	((grain yield per plot (kg) × 10,000 m^2^ ha^−1^)/(effective plot area (m^2^)) × 1000 kg t^−1^).
Grain yield per plant (g)^a^	((grain yield (kg ha^−1^) × 1000)/(respective number of plants in a hectare)

^a^ Measurements were determined on a plot basis. The other traits were determined from five randomly selected samples.

### 2.5 Data Analysis

Data were analyzed using SAS with PROC MIXED METHOD = REML. The denominator degree of freedom (DDF) was determined using the Satterthwaite method. Genotype, plant density, and environment were considered fixed while block and replication were considered as random effects. The model used in the data analysis (taking yield as an example) was:

$$\begin{array}{c} Y_{\text{ijklm}}=\mu +G_{i}+D_{j}+L_{k}+{\mathrm{GD}}_{\mathrm{ij}}+{\mathrm{GL}}_{\mathrm{ik}}+{\mathrm{DL}}_{\mathrm{jk}}+{\mathrm{GDL}}_{\mathrm{ijk}}+B_{\text{ljkm}}+R_{\mathrm{mk}}+E_{\text{ijklm}} \end{array}$$

where, Y_ijklm_ is the yield of the ith genotype in the jth plant density, kth environment, lth block, and mth replication; μ is the overall mean; G_i_ is the fixed effect of ith genotype; D_j_ is the fixed effect of jth plant density; L_k_ is the fixed effect of the kth environment; GD_ij_ is the fixed interaction effect of the ith genotype and jth plant density; GL_ik_ is the fixed interaction effect of the ith genotype and kth environment; DL_jk_ is the fixed interaction effect of jth plant density and kth environment; GDL_ijk_ is the fixed three‐way interaction effect of ith genotype, jth plant density, and kth environment; B_ljkm_ is the random effect of the lth block within jth plant density, kth environment, and mth replication; R_mk_ is the random effect of the mth replication within the kth environment; and E_ijklm_ is the experimental error.

The data for percent root lodging, percent stem lodging, and percent barren plants were transformed with arc sin square root—Arc Sine (Y)^1/2^.

Whenever significant differences were detected, pair‐wise comparisons of the least square means were carried out using PDIFF of the LSMEANS statement in SAS which gives the “*p*” value for differences of LS‐means together with standard error of the difference and *t*‐value. From the pairwise comparisons, only the comparisons of the performance of a given hybrid at the three plant densities were presented. This is to show if a given hybrid was tolerant to high plant density or not. The least square means of a trait for each plant density was presented, and similar letters were assigned to the least square means when the difference between the pair was not significant.

## 3 Results

### 3.1 Performance of Vegetative, Phenology, and Tassel Related Traits of Hybrids Under Three Plant Densities Across Four Environments

Plant density (PD) significantly affected most of the traits of the hybrids including ear height (EH), plant height to ear height ratio (PH/EH), anthesis‐siking‐interval (ASI), percent root lodging (RL), leaf angle (LA), stem diameter (SD), tassel size (TS), and chlorophyll content (ChC). However, the effect of plant density was not significant for plant height (PH), days to 50% anthesis (DA), days to 50% silking (DS), percent stem lodging (SL), and upper leaf number (ULN) (Table 4). Environment (E) was also significant for most of the traits except in root lodging, stem lodging, and the tassel‐related traits of the hybrids. Genotype × plant density interaction was significant only for ear height and plant height to ear height ratio. Genotype × environment interaction significantly affected all of the vegetative and phenology‐related traits tested. Plant density × environment interaction significantly affected most of the traits tested except days to 50% anthesis, days to 50% silking, and percent stem lodging of the hybrids. However, the three‐way interaction of genotype, plant density, and environment was significant only for ear height, and plant height to ear height ratio of the hybrids. Days to maturity (DM), which was recorded only in Legon (off‐season), was significant only for genotype but neither significant for plant density nor genotype × plant density interaction (Table 4).

**TABLE 4:** Effects of genotype, plant density, and environment on vegetative, phenology, and tassel related traits of maize hybrids.

Source/trait	PH			EH			PH/EH			RL			SL			ULN			LA		
	NDF	DDF	*p*	NDF	DDF	*p*	NDF	DDF	*p*	NDF	DDF	*p*	NDF	DDF	*p*	NDF	DDF	*p*	NDF	DDF	*p*
G	47	448	**	47	459	**	47	495	**	47	493	**	47	538	**	47	541	**	47	526	**
PD	2	147	NS	2	139	**	2	495	**	2	132	**	2	82	NS	2	62	NS	2	97	*
E	3	4	*	3	4	**	3	495	**	3	4	NS	3	4	NS	3	4	*	3	4	**
G × PD	94	446	NS	94	456	**	94	495	**	94	489	NS	94	530	NS	94	529	NS	94	520	NS
G × E	141	443	**	141	453	**	141	495	**	141	485	**	141	515	*	141	506	**	141	509	**
PD × E	6	147	*	6	139	**	6	495	**	6	132	*	6	82	NS	6	62	*	6	97	**
G × PD × E	282	439	NS	282	448	**	282	495	**	282	479	NS	282	500	NS	282	482	NS	282	501	NS

Source/trait	SD			TS			CHC			DA			DS			ASI			DM		
	NDF	DDF	*p*	NDF	DDF	*p*	NDF	DDF	*p*	NDF	DDF	*p*	NDF	DDF	*p*	NDF	DDF	*p*	NDF	DDF	*p*
G	47	497	**	47	398	**	47	254	**	47	484	**	47	492	**	47	504	**	47	116	**
PD	2	114	**	2	56	**	2	48	**	2	119	NS	2	118	NS	2	104	**	2	24	NS
E	3	4	**	2	3	NS	1	2	*	3	4	**	3	4	**	3	4	**			
G × PD	94	493	NS	94	390	NS	94	249	NS	94	480	NS	94	488	NS	94	499	NS	94	113	NS
G × E	141	486	**	94	390	**	47	254	*	141	475	**	141	482	**	141	491	**			
PD × E	6	114	**	4	56	**	2	48	**	6	119	NS	6	118	NS	6	104	**			
G × PD × E	282	480	NS	188	377	NS	94	249	NS	282	468	NS	282	476	NS	282	485	NS			

Abbreviations: ASI , anthesis‐silking interval; CHC, chlorophyll content; DA, days to 50% anthesis; DDF, denominator degree of freedom; DM, days to maturity; DS, days to 50% silking; E, environment; EH, ear height; G, genotype; LA, leaf angle; NDF, numerator degree of freedom; NS, not significant (*p* ≥ 0.05); *p*, probability value (i.e., Pr > F); PD, plant density; PH, plant height; PH/EH, plant height to ear height ratio; RL, root lodging; SD, stem diameter; SL, stem lodging; TS, tassel size; ULN, number of upper leaves per plant.

**p < 0.01, *p < 0.05.

Ear height, leaf angle, and percent root lodging were significantly higher under the high plant density compared to the other densities (Table 5). Stem diameter and tassel size were significantly higher under the low density than the other densities. The lowest stem diameter, plant height to ear height ratio, and chlorophyll content were recorded under the high plant density. Anthesis‐silking interval was lowest under the low plant density.

**TABLE 5:** Comparison of least square means of vegetative, phenology, and tassel related traits of hybrids among the three plant densities.

Hybrids									
Trait/Density	EH (cm)	ASI (days)	SD (mm)	PH/EH	RL (%)	LA (°)	TBL (cm)	TS	CHC (spad value)
High	92.1^A^	1.2^A^	17.3^C^	2.13^B^	6.7^A^	24.6^A^	20.4^B^	293.5^B^	47.4^B^
Medium	88.1^B^	1.2^A^	18.8^B^	2.21^A^	3.8^B^	23.8^B^	21.2^A^	302.4^B^	51.8^A^
Low	87.0^B^	1.0^B^	19.6^A^	2.22^A^	2.6^B^	23.9^B^	21.5^A^	313.3^A^	51.0^A^

*Note:* Means with the same letter are not significantly different from each other.

Abbreviations: ASI, anthesis‐silking interval; CHC, chlorophyll content; EH, ear height; LA, leaf angle; PH/EH, plant height to ear height ratio; RL, root lodging; SD, stem diameter; TBL, tassel branch length; TS, tassel size.

### 3.2 Yield and Yield Components of Hybrids Under Three Plant Densities Across Four Environments

Genotype, environment, and plant density significantly affected grain yield, grain yield per plant (YPP), ears per plant (EPP), ear length (EL), filled ear length (FEL), ear diameter (ED), kernels per row (KPR), kernels per ear (KPE), hundred kernel weight (HKW), shelling percentage (SP), and percent barren plants (BP) (Table 6). However, rows per ear (RPE) were affected only by genotype. Similarly, the genotype × environment interaction and plant density × environment interaction were also significant for most of the traits including grain yield, ear length, filled ear length, ear diameter, kernels per row, kernels per ear, and shelling percentage. Ears per plant were significantly affected by the interaction of plant density and environment but not the interaction of genotype and environment. Grain yield per plant, rows per ear, and hundred kernel weight were significantly affected by genotype × environment interaction, but plant density × environment interaction was not significant for these traits. Percent barren plants was not affected by any of the two‐way interactions. The genotype × plant density interaction was significant only for grain yield and grain yield per plant. However, the genotype × environment × plant density interaction was not significant for all the traits (Table 6).

**TABLE 6:** Effects of genotype, plant density, and environment on grain yield and yield components of hybrids.

Source/trait	Grain yield			YPP			EPP			EL			FEL			ED		
	NDF	DDF	*p*	NDF	DDF	*p*	NDF	DDF	*p*	NDF	DDF	*p*	NDF	DDF	*p*	NDF	DDF	*p*
G	47	469	**	47	468	**	47	535	**	47	517	**	47	516	**	47	503	**
PD	2	130	**	2	130	**	2	61.7	**	2	93.4	**	2	100	**	2	96.3	**
E	3	4	**	3	4	*	3	4	*	3	4	**	3	4	**	3	4	**
G × PD	94	466	*	94	465	**	94	523	NS	94	511	NS	94	510	NS	94	498	NS
G × E	141	462	**	141	461	**	141	502	NS	141	500	**	141	501	**	141	489	**
PD × E	6	130	**	6	130	NS	6	61.7	*	6	93.4	**	6	100	**	6	96.3	**
G × PD × E	282	456	NS	282	455	NS	282	481	NS	282	493	NS	282	495	NS	282	483	NS

Source/trait	RPE			KPR			KPE			HKW			SP			BP		
	NDF	DDF	*p*	NDF	DDF	*p*	NDF	DDF	*p*	NDF	DDF	*p*	NDF	DDF	*p*	NDF	DDF	*p*
G	47	537	**	47	521	**	47	514	**	47	538	**	47	533	**	47	539	**
PD	2	59.3	NS	2	87.2	**	2	86	**	2	85	**	2	91.6	**	2	71.6	**
E	3	4	NS	3	87.2	**	3	4	**	3	4	**	3	4	**	3	4	*
G × PD	94	524	NS	94	514	NS	94	507	NS	94	530	NS	94	526	NS	94	530	NS
G × E	141	501	**	141	502	**	141	496	**	141	516	**	141	514	**	141	511	NS
PD × E	6	59.3	NS	6	87.2	**	6	86	**	6	85	NS	6	91.6	*	6	71.6	NS
G × PD × E	282	478	NS	282	491	NS	282	488	NS	282	502	NS	282	502	NS	282	493	NS

Abbreviations: BP, percent barren plants; DDF, denominator degree of freedom; E, environment; ED, ear diameter; EL, ear length; EPP, ears per plant; FEL, filled ear length; G, genotype; HKW, hundred kernel weight; KPE, kernels per ear; KPR, kernels per row; NDF, numerator degree of freedom; NS, not significant (*p* ≥ 0.05); *p*, probability value (i.e., Pr > F); PD, plant density; RPE, rows per ear; SP, shelling percentage; YPP, Grain yield per plant.

**p < 0.01, *p < 0.05.

Shelling percentage and percentage of barren plants were significantly higher under the high plant density compared to the medium and low plant densities for the hybrids (Table 7). However, ears per plant, ear length, filled ear length, ear diameter, kernels per row, kernels per ear, and hundred kernel weight were significantly lower under the high plant density compared to the other densities. The shelling percentage and percentage of barren plants were significantly lower under the low density compared to the other densities. However, ears per plant were significantly higher under the low plant density compared to the other densities. The performances of the other traits under the low density were similar to the medium density (Table 7).

**TABLE 7:** Comparison of least square means of yield components of hybrids at three plant densities.

Trait/Density	EPP	HKW (g)	SP (%)	BP (%)	EL (cm)	FEL (cm)	ED (cm)	KPR	KPE
High	0.94^C^	30.3^B^	83.2^A^	5.4^A^	13.8^B^	12.4^B^	42.9^B^	26.7^B^	377.9^B^
Medium	0.98^B^	31.1^A^	82.8^B^	2.8^B^	14.6^A^	13.2^A^	43.8^A^	28.7^A^	407.2^A^
Low	1.00^A^	30.9^A^	82.3^C^	1.1^C^	14.7^A^	13.4^A^	43.8^A^	28.8^A^	411.2^A^

*Note:* Means with the same letter are not significantly different from each other.

Abbreviations: BP, percent barren plants; ED, ear diameter; EL, ear length; EPP, ears per plant; FEL, filled ear length; HKW, hundred kernel weight; KPE, kernels per ear; KPR, kernels per row; RPE, rows per ear; SP, shelling percentage; YPP, grain yield per plant.

When the analysis was carried out for each environment for the hybrids, both genotype and plant density were significant in all four environments (Table 8). However, the genotype × plant density was significant only in Fumesua but not in the other three environments.

**TABLE 8:** Effects of genotype and plant density on grain yield (t ha^−1^) of hybrids in each growing environment.

Environment	G	PD	G × PD
Fumesua			
NDF	47	2	94
DDF	109	35.7	107
Pr > F	< 0.0001	< 0.0001	0.01
Legon (off‐season)			
NDF	47	2	94
DDF	114	35	112
Pr > F	< 0.0001	0.0027	0.8395
Legon (minor season)			
NDF	47	2	94
DDF	111	31.3	109
Pr > F	< 0.0001	< 0.0001	0.0811
Nyankpala			
NDF	47	2	94
DDF	123	23	120
Pr > F	< 0.0001	0.0002	0.2948

Abbreviations: DDF, denominator degree of freedom; E, environment; G, genotype; NDF, numerator degree of freedom; PD, plant density.

At each of the four locations, grain yield for the hybrids was lowest under the low density except at Legon during the off‐season where yield under low and medium densities was not significantly different (Table 9). Grain yield was highest (*p* < 0.05) under high density in three of the locations while in Nyankpala, the fourth environment, yield under the medium density was significantly higher than yield under high density.

**TABLE 9:** Comparison of least square means of grain yield (t ha^−1^) of hybrids among the three plant densities in each of the growing environments.

Environment/Density	Fumesua	Legon (Minor)	Legon (off‐season)	Nyankpala
High	6.9^A^	6.4^A^	6.6^A^	4.6^B^
Medium	6.2^B^	5.7^B^	5.9^B^	5.1^A^
Low	5.5^C^	5.2^C^	5.5^B^	4.3^C^
Range	3.7–9.2	2.4–9.5	3.3–8.6	0.9–7.9
Median	6.3	5.8	6	4.9

*Note:* Means with the same letter along the column are not significantly different from each other.

When the grain yield of each hybrid under one plant density was compared to the respective yields under the other two densities, all the top 10 hybrids had significantly higher yields either in the high or medium densities than the low density in Fumesua (Table 10). The highest grain yield in nine of the top 10 hybrids was recorded under the high density but some of them were not significantly different from the medium density. CML16 × 87,036, the highest yielding hybrid in Fumesua had a significantly higher grain yield (9.2 t ha^−1^) under high density than the low density (7.5 t ha^−1^) but the yield of the medium density was intermediate (8.3 t ha^−1^). Similarly, the second highest yielding hybrid in Fumesua, CML16 × 1368, had a significantly higher grain yield (9 t ha^−1^) under the high density than the medium density (7.0 t ha^−1^) and low density (6.2 t ha^−1^). The highest yielding hybrid in Legon (minor season), M131 × CML16, produced a significantly higher grain yield (9.5 t ha^−1^) under high density than under the low density (7.5 t ha^−1^) but the yield of the medium density was intermediate (8.2 t ha^−1^). Similarly, the second highest yielding hybrid, CML16 × ENT11, produced a significantly higher grain yield (8.4 t ha^−1^) under the high density than the medium density (6.6 t ha^−1^) and the low plant density (6.1 t ha^−1^) in Legon (minor season). However, the response of the top‐yielding hybrids to differences in plant density in Legon (off‐season) was lower than in Fumesua and Legon (minor season). Among the top four hybrids, only the highest‐yielding hybrid (ENT11 × 87,036) had a significantly higher grain yield (8.6 t ha^−1^) under the high plant density than under low plant density (6.6 t ha^−1^) in Legon (off‐season) although the means were always higher under the high plant density (Table 10). The highest yielding hybrid in Nyankpala (CML16 × 87,036) had a significantly higher yield (7.9 t ha^−1^) under the medium density than the high density (5.0 t ha^−1^) and low density (5.3 t ha^−1^). Only one hybrid (TZMI740 × CML16), among the top ten hybrids, had a significantly higher grain yield (6.3 t ha^−1^) under the high density than the low density (4.3 t ha^−1^) in Nyankpala. Seven of the hybrids, among the top 10 highest yielding hybrids, in Nyankpala did not show yield differences across the three plant densities (Table 10).

**TABLE 10:** Comparisons of grain yield (t ha^−1^) of top ten hybrids among the three plant densities in each of the growing environments.

Fumesua			Legon (off‐season)			Legon (minor season)			Nyankpala		
Genotype^a^	PD	LSM (t ha^−1^)	Genotype	PD	LSM (t ha^−1^)	Genotype	PD	LSM (t ha^−1^)	Genotype	PD	LSM (t ha^−1^)
CML16 × 87,036	H	9.2^A^	ENT11 × 87,036	H	8.6^A^	M131 × CML16	H	9.5^A^	CML16 × 87,036	H	5.0^B^
	M	8.3^AB^		M	7.0^AB^		M	8.2^AB^		M	7.9^A^
	L	7.5^B^		L	6.6^B^		L	7.5^B^		L	5.3^B^
CML16 × 1368	H	9.0^A^	M131 × CML16	H	8.5^A^	CML16 × ENT11	H	8.4^A^	M131 × CML16	H	5.6^A^
	M	7.0^B^		M	6.9^A^		M	6.6^B^		M	6.7^A^
	L	6.2^B^		L	6.9^A^		L	6.1^B^		L	7.1^A^
TZEI1 × 87,036	H	8.1^A^	CML16 × TZEI1	H	8.4^A^	CML16 × 87,036	H	7.0^A^	PAN53	H	6.9^A^
	M	8.7^A^		M	7.8^A^		M	8.0^A^		M	7.0^A^
	L	6.0^B^		L	6.7^A^		L	5.1^B^		L	5.5^A^
ENT11 × 87,036	H	8.5^A^	CML16 × 87,036	H	8.4^A^	TZdEI501 × ENT11	H	7.1^AB^	ENT11 × 87,036	H	5.1^B^
	M	7.5^AB^		M	7.8^A^		M	6.2^B^		M	6.7^A^
	L	7.1^B^		L	7.3^A^		L	7.9^A^		L	5.0^B^
M131 × CML16	H	8.3^A^	TZdEI501 × ENT11	H	8.3^A^	M131 × EXP124	H	7.7^A^	M131 × TZEI7	H	4.1^B^
	M	6.9^B^		M	5.9^B^		M	5.0^B^		M	6.3^A^
	L	6.5^B^		L	5.7^B^		L	5.8^B^		L	5.0^AB^
1368 × 87,036	H	8.3^A^	TZdEI525 × M131	H	8.1^A^	CML16 × TZEI1	H	7.4^A^	M131 × ENT11	H	5.1^AB^
	M	7.6^A^		M	7.0^AB^		M	6.6^AB^		M	6.3^A^
	L	6.0^B^		L	5.5^B^		L	5.5^B^		L	4.2^B^
M131 × TZdEI501	H	8.2^A^	M131 × ENT11	H	7.9^A^	TZdEI501 × CML16	H	7.4^A^	TZMI740 × CML16	H	6.3^A^
	M	7.0^A^		M	7.5^A^		M	6.2^AB^		M	5.9^A^
	L	5.4^B^		L	6.3^A^		L	5.6^B^		L	4.3^B^
PAN53	H	8.0^A^	TZMI740 × ENT11	H	7.8^A^	ENT11 × 87,036	H	7.3^A^	M131 × TZEI387	H	5.4^A^
	M	6.7^B^		M	5.6^B^		M	6.4^A^		M	6.0^A^
	L	7.3^AB^		L	5.6^B^		L	6.5^A^		L	4.5^A^
CML16 × TZEI1	H	7.9^A^	CML16 × TZEI387	H	7.7^A^	M131 × ENT11	H	7.2^A^	CML16 × TZEI7	H	5.4^A^
	M	6.6^B^		M	4.9^B^		M	5.6^B^		M	5.9^A^
	L	6.1^B^		L	5.9^B^		L	5.8^B^		L	5.1^A^
TZMI740 × 1368	H	7.9^A^	TZEI1 × 87,036	H	7.5^A^	CML16 × TZEI7	H	7.2^A^	TZdEI501 × CML16	H	4.9^A^
	M	6.0^B^		M	6.1^A^		M	6.3^A^		M	5.9^A^
	L	5.2^B^		L	6.3^A^		L	6.1^A^		L	5.3^A^

*Note:* Means with the same letter along the column are not significantly different from each other.

Abbreviations: H, high plant density; L, low plant density; LSM, least square mean; M, medium plant density; PD, plant density.

^a^ Hybrids are selected based on their highest grain yield in any of the three plant densities in each environment.

## 4 Discussion

### 4.1 Response of Vegetative, Phenology, and Tassel Related Traits of Hybrids to Differences in Plant Density and Environment

The non‐significant interaction effect of genotype and plant density for most of the morphological and phenology‐related traits and the non‐significant three‐way interaction effects for all these traits implies the relative performance of the hybrids for these traits was not dependent on plant density. The significant interaction effect of genotype and environment implies the relative performance of the genotypes for these traits was dependent on the growing environment. The significant effect of genotype on almost all the vegetative, phenology, and tassel related traits tested except the tassel branch number implies the hybrids were genetically diverse for the vegetative, phenology, and tassel‐related traits tested.

The non‐significant effect of plant density on plant height, in the current study, contradicts the findings of other researchers who have reported an increased plant height with an increase in plant density (Al‐Naggar et al. 2017; Brekke et al. 2011; Dawadi and Sah 2012; Mansfield and Mumm 2014; Nwogboduhu 2016; Sangoi and Salvador 1998). Generally, plant height increases with an increase in plant density. This is a mechanism that plants use to avoid being shaded by neighboring plants (Ford 2014). Crowded planting might reduce the ratio of red to far red radiations that reach the plant leaves, and this, in turn, initiates an increased apical dominance (Holalu and Finlayson 2017) which causes an increase in plant height especially in herbaceous plants (Ford 2014). However, under low plant density, there is a possibility of increased far‐red radiation from horizontal reflection of light by the lower plant parts of neighboring plants which generally receive more light than under high density. Therefore, the plants under low density which are exposed to horizontally reflected light could also grow taller depending on the proportion of the red and far‐red radiations (Ford 2014). Similarly, plant height is also affected by the availability of soil nutrients, especially nitrogen. Plant height at supra‐optimum density was shorter than the plant height under the low densities when nitrogen fertilizer was not applied (Boomsma et al. 2009). Thus, the effect of plant density on plant height is dependent on many other conditions including the nutrient status of the soil and the quality and intensity of the solar radiation. In the current study, the same amount of fertilizer was applied to all plots regardless of plant density, which means individual plants under the high plant density received lower amount of fertilizer than their counterparts under the low density. Therefore, it is possible that the lower amount (on plant basis) of fertilizer, especially nitrogen in the high density compromised the effect of shading on increasing plant height. Similar to the current findings, Biswas et al. (2014) have reported that plant height did not change with increased plant density. Brekke et al. (2011) also reported no change in plant height with increased plant density for maize plants from an unselected population.

The increased ASI with increased plant density in the current study agrees with the findings of Sangoi et al. (2002) and Al‐Naggar et al. (2017). However, Brekke et al. (2011) reported an increase in ASI with an increase in plant density in the unselected or less advanced selected population but not in the more advanced selected population. The non‐significant effect of plant density on leaf angle in the current study is in contradiction to the findings of Al‐Naggar et al. (2017) who reported that leaf angle decreased with an increase in plant density. However, Brekke et al. (2011) did not find any differences in leaf angle among plant densities. It was also reported that, generally, leaves of modern maize hybrids tend to be more upright and thus the shading effect of their leaves on the neighboring plants is minimized (Ford 2014). However, plants can also tend to shade neighboring plants by expanding their leaves so as to suppress and outcompete the neighboring plants. The function of the leaves of plants when there is crowding is to photosynthesize and at the same time to shade neighboring plants (Ford 2014). Thus, in the current study, the tendency for shading neighboring plants might have caused the slight increase (2.9%) in leaf angle under high density. This indicates improvement in the erectness of the leaves of the hybrids is needed in order to make them more suitable for high‐density planting.

### 4.2 Response of Yield and Yield Components of Hybrids to Differences in Plant Density and Growing Environment

Significant genotype × plant density interaction for grain yield of the hybrids implies that the hybrids differed in their response to changes in plant densities. Similarly, significant plant density × environment interaction indicates the optimal plant density for the hybrids differed with growing environments. Moreover, significant genotype × environment interaction denotes the relative performance of hybrids differed with environment. This signifies the need for selecting specific superior hybrids for specific growing environments. This is in line with the findings of Gebre (2005) who reported significant genotype and environment interaction in maize hybrids. Similar findings were reported by Seyoum et al. (2019) and Mansfield and Mumm (2014) where the interactions of genotype and density were significant in maize hybrids.

The interaction effect of genotype and plant density was, generally, significant in the combined analysis. However, when a separate analysis was made for each environment, the interaction effect of genotype and plant density was significant in Fumesua but not in the other environments. This implies the relative grain yield performances of the hybrids in the other three environments were not dependent on plant density. When the grain yield of each of the top ten hybrids at one plant density was compared to the other two respective densities within each environment, the hybrids showed a differential response to plant density. For example, in Fumesua, the grain yield of CML16 × 87,036 was significantly higher under the high plant density than the low density but there was no difference in the yield of this hybrid at medium and low densities. The grain yield of M131 × CML16 was significantly higher under the high density than the medium and low densities while the yield under the medium density was also higher than the low density. This means M131 × CML16 showed a significant yield increase both when the density changed from low to medium and from medium to high. However, the grain yield of CML16 × 87,036 significantly increased only when the density was changed from low to high, indicating the differential yield response of the genotypes to differences in plant density. Similarly, in the other three environments, some hybrids did not show variations in grain yield with changes in plant density while others showed significant variation in grain yield across plant densities which also indicated the differential response of the hybrids to plant densities.

The better grain yield performance under the high density than the low density for most of the top ten hybrids in Fumesua and some of the top ten hybrids in Legon (off‐season) and Legon (minor season) might imply the suitability of these environments to high‐density planting. Moreover, the highest grain yield in each of these environments was also obtained from the high‐density planting which indicated the potential of these environments, especially Fumesua, to support high‐density planting. Most of the top ten hybrids in Nyankapala did not respond to variations in density but some of them were better under the medium density compared to the high and low densities. This could imply that Nyankpla was not productive enough to support the growth of more crowded maize plants in comparison to the other three environments. The soils in Nyankpala were relatively more acidic compared to other environments, and thus, this might hinder phosphorus absorption by plants (Tandzi et al. 2018) although the recommended fertilizer per unit area was applied. Moreover, the organic carbon content and cation exchange capacity (CEC) of the soil of Nyankpala were also very low compared to the other environments. Therefore, it could be because of these limitations in soil nutrients that Nyankpala was not productive enough for high‐density planting, but it was suitable for the medium density.

It was reported that in general maize yield increased with an increase in plant density up to a certain limit but it declined with further increase of plant density except for very marginal conditions (arid environments) where maize may not respond to variations in plant density (Haarhoff and Swanepoel 2018). The peak might go up to 120,000 to 150,000 plants ha^−1^ depending on the genotypes and growing environments (Haarhoff and Swanepoel 2018; Kosmelj et al. 2005). This implies that the hybrids which performed best under the high plant density, in the current study, might or might not have reached the peak plant density beyond which their yields would decline. The same amount of fertilizer per hectare was applied to all three densities. Moreover, the yield variability of the hybrids in response to plant density implies the importance of determining the optimum planting density for a given maize hybrid rather than using blanket recommendations. The interaction of genotype and plant density was non‐significant for almost all the yield components indicating the relative performances of the hybrids for these yield components did not show variations across plant densities.

The reduction in the performance of the yield components such as ears per plant, number of kernels per ear, and hundred kernel weight with increasing plant density were in line with the findings of Al‐Naggar et al. (2017). Similar to the findings of the current study, Sangoi et al. (2002) reported an increase in the percent of barren plants with an increase in plant density. The reduction in the performance of most of the yield components and the increased of percentage barren plants under the high plant density could be because of the competition among the individual plants for limited resources. Under increased plant densities, inter‐plant competition may cause the suppression of some plants by others, and the dominant plants will grow taller and shade the suppressed plants (Rossini et al. 2011). In the current study, the increased shelling percentage with increased plant density implies the high plant density had a more drastic effect on the cob than on the kernels.

Farmers in Sub‐Saharan Africa could boost their maize yield by adopting hybrids that have superior performance under high plant density. Thus, policymakers in Sub‐Saharan Africa should consider high plant density‐tolerant maize hybrids as a means for increasing productivity and encourage studies on improving high plant density tolerance of maize.

Among the limitations of the current study is the use irrigation in some experiments when most of the small‐scale farmers in Africa do not have access to irrigation. Days to maturity, chlorophyll content, and tassel size were determined only from one, two, and three environments, respectively. Moreover, the economic trade‐off between the increase in yield and cost of high‐density planting was not examined. High plant density requires more labor for planting and fertilizer application. It also requires more seeds, but it might need less labor for weeding as high‐density planting might suppress weeds. Therefore, further study of selected promising hybrids under more than three plant densities would provide more comprehensive understanding of the optimum plant density. Similarly, evaluations of selected superior hybrids under different fertilizer levels across different plant densities would offer better information on the potential yield of hybrids under different plant densities. The cost‐benefit analysis of high plant density should also be studied in the future.

## 5 Conclusion

High plant density significantly reduced important yield components such as ear per plant, ear length, filled ear length, ear diameter, kernel per ear, and kernel per ear. However, the shelling percentage and percentage of barren plants were significantly high in the high plant density. The relative grain yield performance of the hybrids was dependent on plant density and on the growing environment. Similarly, the optimum plant density for the hybrids varied with growing environments. The highest grain yield in each environment was obtained from the high density except in Nyankpala where the highest yield was obtained from the medium density. A yield increase between 22.7% and 30% was obtained from the highest‐yielding hybrids under high plant density in the high‐yielding environments compared to the respective yield under the low plant density. The increased number of plants per hectare in the high density compensated for the reduction in the ear per plant, ear length, filled ear length, ear diameter, kernel per row, and kernel per ear accounting for the high grain yield. This could also be attributed to the hybrids ability to tolerate high plant density which in this study is 88,888 plants ha^−1^. Generally, high‐yielding environments were suitable for high plant density while low‐yielding environments for medium plant density. F_1_ hybrids M131 × CML116, CML16 × TZEI1, CML16 × 87,036, TZEI387 × CML16, and ENT11 × 87,036 are good candidates for high‐density planting in high‐yielding environments.

## Conflicts of Interest

The authors declare no conflicts of interest.

## Data Availability

The data that support the findings of this study are openly available in Dryad at https://doi.org/10.5061/dryad.sbcc2frj9.

## References

[pei370046-bib-0001] Abbam, T. , F. A. Johnson , J. Dash , and S. S. Padmadas . 2018. “Spatiotemporal Variations in Rainfall and Temperature in Ghana Over the Twentieth Century, 1900–2014.” Earth and Space Science 5: 120–132. 10.1002/2017EA000327.

[pei370046-bib-0002] AccuWeather . 2019. “Accra, Greater Accra, Ghana Monthly Weather.” https://www.accuweather.com/en/gh/accra/178551/january‐weather/178551?year=2018&view=list.

[pei370046-bib-0003] Adu, G. B. , M. S. Abdulai , H. Alidu , et al. 2014. Recommended Production Practices for Maize in Ghana. AGRA/CSIR. 10.13140/2.1.4376.3527.

[pei370046-bib-0004] Ajayo, B. , B. Badu‐Apraku , M. Fakorede , and R. Akinwale . 2021. “Plant Density and Nitrogen Responses of Maize Hybrids in Diverse Agro‐Ecologies of West and Central Africa.” AIMS Agriculture and Food 6, no. 1: 381–400.

[pei370046-bib-0005] Alkhalifah, N. B. 2013. “Density Response of Maize Canopy Architecture in Adapted and Unadapted Synthetic Populations. Theses and Dessertation, Iowa State University, USA.”

[pei370046-bib-0006] Al‐Naggar, A. M. M. , M. M. M. Atta , M. A. Ahmed , and A. S. M. Younis . 2016. “Genetic Variance, Heritability and Selection Gain of Maize (*Zea mays* L.) Adaptive Traits to High Plant Density Combined With Water Stress.” Journal of Applied Life Sciences International 7, no. 2: 1–17. 10.9734/JABB/2016/28603.

[pei370046-bib-0007] Al‐Naggar, A. M. M. , R. A. Shabana , M. S. Hassanein , and A. M. A. Metwally . 2017. “Effects of Genotype, Plant Density and Their Interaction on Maize Yield and Traits Related to Plant Density Tolerance.” Bioscience Research 14, no. 2: 395–407.

[pei370046-bib-0008] Aramburu‐Merlos, F. , F. A. Tenorio , N. Mashingaidze , et al. 2024. “Adopting Yield‐Improving Practices to Meet Maize Demand in Sub‐Saharan Africa Without Cropland Expansion.” Nature Communications 15, no. 1: 4492. 10.1038/s41467-024-48859-0. PMC11130130 38802418

[pei370046-bib-0009] Assefa, Y. , P. Carter , M. Hinds , et al. 2018. “Analysis of Long Term Study Indicates Both Agronomic Optimal Plant Density and Increase Maize Yield per Plant Contributed to Yield Gain.” Scientific Reports 8: 4937. 10.1038/s41598-018-23362-x. 29563534 PMC5862987

[pei370046-bib-0010] Biswas, M. , A. Rahman , and F. Ahmed . 2014. “Effect of Variety and Planting Geometry on the Growth and Yield of Hybrid Maize.” Journal of Agriculture and Environmental Sciences 3, no. 2: 27–32.

[pei370046-bib-0011] Boomsma, C. R. , J. B. Santini , M. Tollenaar , and T. J. Vyn . 2009. “Maize Morphophysiological Responses to Intense Crowding and Low Nitrogen Availability: An Analysis and Review.” Agronomy Journal 101, no. 6: 1426–1452. 10.2134/agronj2009.0082.

[pei370046-bib-0012] Brekke, B. , J. Edwards , and A. Knapp . 2011. “Selection and Adaptation to High Plant Density in the Iowa Stiff Stalk Synthetic Maize ( *Zea mays* L.) Population.” Crop Science 51: 1965–1972. 10.2135/cropsci2010.09.0563.

[pei370046-bib-0013] Buah, S. S. J. , L. N. Abatania , and G. K. S. Aflakpui . 2009. “Quality Protein Maize Response to Nitrogen Rate and Plant Density in the Guinea Savanna Zone of Ghana.” West Africa Journal of Applied Ecology 16: 9–21.

[pei370046-bib-0014] Dawadi, D. R. , and S. K. Sah . 2012. “Growth and Yield of Hybrid Maize (*Zea mays* L.) in Relation to Planting Density and Nitrogen Levels During Winter Season in Nepal.” Tropical Agricultural Research 23, no. 3: 218–227.

[pei370046-bib-0015] Duvick, D. N. 2005. “The Contribution of Breeding to Yield Advances in Maize ( *Zea mays* L.).” Advances in Agronomy 86: 83–145.

[pei370046-bib-0016] FAOSTAT . 2024. Crop Production Statistics. Food and Agriculture Organization. https://www.fao.org/faostat/en/#data/QCL.

[pei370046-bib-0017] Fischer, T. , D. Byerlee , and G. Edmeades . 2014. “Crop Yields and Global Food Security: Will Yield Increase Continue to Feed the World? ACIAR Monograph No. 158. Australian Centre for International Agricultural Research.”

[pei370046-bib-0018] Ford, E. D. 2014. “The Dynamic Relationship Between Plant Architecture and Competition.” Frontiers in Plant Science 5: 1–13. 10.3389/fpls.2014.00275. PMC4060642 24987396

[pei370046-bib-0019] Gebre, G. B. 2005. “Genetic Variability and Inheritance of Drought and Plant Density Adaptive Traits in Maize. PhD Thesis Desertation, University of the Free State, South Africa.”

[pei370046-bib-0020] Ghana Meteorological Agency (GMet) . 2019. Climatological Services. Accra.

[pei370046-bib-0021] Ghana Statistical Service . 2014a. 2010 Population & Housing Census: District Analytical Report: Kumasi Metroppolitan. Ghana Statistical Service.

[pei370046-bib-0022] Ghana Statistical Service . 2014b. 2010 Population & Housing Census: District Analytical Report: Tolon District. Ghana Statistical Service.

[pei370046-bib-0023] Haarhoff, S. J. , and P. A. Swanepoel . 2018. “Plant Population and Maize Grain Yield: A Global Systematic Review of Rainfed Trials.” Crop Science 58: 1819–1829. 10.2135/cropsci2018.01.0003.

[pei370046-bib-0024] Holalu, S. V. , and S. A. Finlayson . 2017. “The Ratio of Red Light to Far Red Light Alters Arabidopsis Axillary Bud Growth and Abscisic Acid Signalling Before Stem Auxin Changes.” Journal of Experimental Botany 68, no. 5: 943–952. 10.1093/jxb/erw479. 28062593 PMC5444464

[pei370046-bib-0025] Kamara, A. Y. , A. Menkir , A. W. Abubakar , et al. 2020. “Maize Hybrids Response to High Plant Density in the Guinea Savannah of Nigeria.” Journal of Crop Improvement 35, no. 1: 1–20.

[pei370046-bib-0026] Kosmelj, K. , A. Blejec , and A. Cedilnik . 2005. “Interval Estimate for Specific Points in Polynomial Regression.” Journal of Computing and Information Technology‐CIT 13, no. 4: 287–291.

[pei370046-bib-0027] Kwame Nkrumah University of Science and Technology (KNUST) . 2019. Weather Records for 2018. Kumasi.

[pei370046-bib-0028] Mansfield, B. D. , and R. H. Mumm . 2014. “Survey of Plant Density Tolerance in U. S. Maize Germplasm.” Crop Science 54: 157–173. 10.2135/cropsci2013.04.0252.

[pei370046-bib-0029] Matthijs, T. , and J. Wu . 2000. Physiological Bases for Crop Improvement, edited by M. E. Otegui and G. A. Slafer . Food Products Press, an Imprint of the Haworth Press, Inc.

[pei370046-bib-0030] Ministry of Food and Agriculture (MoFA) . 2016. “Agriculture in Ghana. Facts and Figures (2015). Issued by Statistics, Research and Information Directorate. Accra, Ghana.”

[pei370046-bib-0031] Ministry of Food and Agriculture (MoFA) . 2021. “Facts and Figures: Agriculture in Ghana, 2020. Issued by Statistics, Research and Information Directorate. Acra, Ghana.”

[pei370046-bib-0032] Nkrumah, F. , N. A. B. Klutse , D. C. Adukpo , et al. 2014. “Rainfall Variability Over Ghana: Model Versus Rain Gauge Observation.” International Journal of Geosciences 5: 673–683. 10.4236/ijg.2014.57060.

[pei370046-bib-0033] Nwogboduhu, N. G. 2016. “Response of Maize (*Zea mays* L.) Varieties to Planting Densities.” IOSR Journal of Agriculture and Veterinary Science 9, no. 10: 1–6. 10.9790/2380-0910010106.

[pei370046-bib-0034] Rossini, M. A. , G. A. Maddonni , and M. E. Otegui . 2011. “Inter‐Plant Competition for Resources in Maize Crops Grown Under Contrasting Nitrogen Supply and Density: Variability in Plant and Ear Growth.” Field Crops Research 121: 373–380. 10.1016/j.fcr.2011.01.003.

[pei370046-bib-0035] Sangoi, L. 2000. “Understanding Plant Density Effects on Maize Growth and Development: An Important Issue to Maximize Grain Yield.” Ciência Rural 31, no. 1: 159–168.

[pei370046-bib-0036] Sangoi, L. , M. Ender , A. F. Guidolin , M. L. de Almeida , and e. P. C. Heberle . 2001. “Influence of Row Spacing Reduction on Maize Grain Yield in Regions With a Short Summer.” Pesquisa Agropecuária Brasileira 36, no. 6: 861–869.

[pei370046-bib-0037] Sangoi, L. , M. A. Gracietti , C. Rampazzo , and P. Bianchetti . 2002. “Response of Brazilian Maize Hybrids From Different Eras to Changes in Plant Density.” Field Crops Research 79: 39–51.

[pei370046-bib-0038] Sangoi, L. , and R. J. Salvador . 1998. “Influence of Plant Height and of Leaf Number on Maize Production at High Plant Densities.” Pesquisa Agropecuária Brasileira 33: 297–306.

[pei370046-bib-0039] Savanna Agricultural Research Institute (SARI) . 2019. “Weather Record for Nyankpala Station, Tamale, Ghana. Tamale, Ghana.”

[pei370046-bib-0040] Seyoum, S. , R. Rachaputi , S. Fekybelu , Y. Chauhan , and B. Prasanna . 2019. “Exploiting Genotype × Environment × Management Interactions to Enhance Maize Productivity in Ethiopia.” European Journal of Agronomy 103: 165–174. 10.1016/j.eja.2018.12.011.

[pei370046-bib-0041] Sharifi, R. S. M. S. , and A. Gholipouri . 2009. “Effect of Population Density on Yield and Yield Attributes of Maize Hybrids.” Research Journal of Biological Sciences 4, no. 4: 375–379.

[pei370046-bib-0042] Sibonginkosi, N. , M. Mzwandile , and T. Tamado . 2020. “Effect of Plant Density on Growth and Yield of Maize [*Zea mays* (L.)] Hybrids at Luyengo, Middleveld of Eswatini.” Asian Plant Research Journal 3, no. 3–4: 1–9. 10.9734/APRJ/2019/v3i3-430066.

[pei370046-bib-0043] Tandzi, L. N. , C. S. Mutengwa , E. L. M. Ngonkeu , and V. Gracen . 2018. “Breeding Maize for Tolerance to Acidic Soils: A Review.” Agronomy 8, no. 6: 1–21. 10.3390/agronomy8060084.

[pei370046-bib-0044] Tontoh, A. A. 2011. “A Study to Ascertain the Growth Situation of the Kumasi Metropolitan Area (KMA): A Remote Sensing Approach. MSc Thesis Desertation, Kwame Nkrumah University of Science and Technology, Kumasi, Ghana.”

[pei370046-bib-0045] Verheye, W. 2010. “Growth and Production of Maize: Traditional Low‐Input Cultivation.” In Land Use, Land Cover and Soil Sciences, edited by W. Verheye . Encyclopedia of Life Support Systems (EOLSS), UNESCO‐EOLSS Publishers.

[pei370046-bib-0046] Worku, A. , B. Derebe , Y. Bitew , G. Chakelie , and M. Andualem . 2020. “Response of Maize (*Zea mays* L.) to Nitrogen and Planting Density in Jabitahinan District, Western Amhara Region.” Cogent Food & Agriculture 6, no. 1: 1770405. 10.1080/23311932.2020.1770405.

